# Deformed Wing Virus in Two Widespread Invasive Ants: Geographical Distribution, Prevalence, and Phylogeny

**DOI:** 10.3390/v12111309

**Published:** 2020-11-15

**Authors:** Chun-Yi Lin, Chih-Chi Lee, Yu-Shin Nai, Hung-Wei Hsu, Chow-Yang Lee, Kazuki Tsuji, Chin-Cheng Scotty Yang

**Affiliations:** 1 Research Institute for Sustainable Humanosphere, Kyoto University, Kyoto 611-0011, Japan; chunyitonylin@gmail.com (C.-Y.L.); lee.chihchi.54m@st.kyoto-u.ac.jp (C.-C.L.); max7297@gmail.com (H.-W.H.); 2 Laboratory of Insect Ecology, Graduate School of Agriculture, Kyoto University, Kyoto 606-8502, Japan; 3 Department of Entomology, National Chung Hsing University, Taichung 402204, Taiwan; ysnai@nchu.edu.tw; 4 Department of Entomology, University of California, 900 University Avenue, Riverside, CA 92521, USA; chowyang.lee@ucr.edu; 5 Department of Subtropical Agro-Environmental Sciences, University of the Ryukyus, Senbaru 1, Nishihara, Okinawa 903-0213, Japan; tsujik@agr.u-ryukyu.ac.jp; 6 Department of Entomology, Virginia Polytechnic Institute and State University, Blacksburg, VA 24061, USA

**Keywords:** deformed wing virus, honey bee, longhorn crazy ant, virus spillover, yellow crazy ant

## Abstract

Spillover of honey bee viruses have posed a significant threat to pollination services, triggering substantial effort in determining the host range of the viruses as an attempt to understand the transmission dynamics. Previous studies have reported infection of honey bee viruses in ants, raising the concern of ants serving as a reservoir host. Most of these studies, however, are restricted to a single, local ant population. We assessed the status (geographical distribution/prevalence/viral replication) and phylogenetic relationships of honey bee viruses in ants across the Asia–Pacific region, using deformed wing virus (DWV) and two widespread invasive ants, *Paratrechina longicornis* and *Anoplolepis gracilipes*, as the study system. DWV was detected in both ant species, with differential geographical distribution patterns and prevenance levels between them. These metrics, however, are consistent across the geographical range of the same ant species. Active replication was only evident in *P*. *longicornis*. We also showed that ant-associated DWV is genetically similar to that isolated from Asian populations of honey bees, suggesting that local acquisition of DWV by the invasive ants may have been common at least in some of our sampled regions. Transmission efficiency of DWV to local arthropods mediated by ant, however, may vary across ant species.

## 1. Introduction

Invasive species are a well-recognized source of threat to native communities and have caused a significant loss of biodiversity on a global scale [1]. One of the accompanying risks is that some of these invasive species may serve as a reservoir of pathogens when introduced to a new environment. Such novel, newly arriving pathogens can lead to devastating consequences for native species [2]. A notable example is that a parapoxvirus, introduced along with the grey squirrel (*Sciurus carolinensis*), was shown to contribute to the significant decline of native squirrels (*S*. *vulgaris*) in the United Kingdom [3]. As the pathogen-mediated competition hypothesis predicts, the invasive species may benefit from their ability to tolerate pathogens, thus rendering them selectively advantageous over their native competitors [4].

Pathogen spillover occurs when a pathogen is introduced and transmitted from a reservoir population into a naive host population [5]. Pathogen spillover following the introduction of an invasive species may enhance the spread of the pathogen itself and, sometimes, the speed of invasion of its host [6,7]. One striking example is that an ectoparasitic mite, *Varroa destructor*, has been found to act as a viral vector that significantly increases the prevalence of deformed wing virus (DWV) up to 100% within populations of the European honey bee (*Apis mellifera*) in Hawaii [8]. Furthermore, recent studies have shown that the honey bee viral pathogens could be detected in various pollinating insect groups, including bumblebees, other Hymenopteran insects, and hoverflies. These viruses can be effectively transmitted via ecological interactions among the pollinators [9,10,11,12]. Figueroa et al. [13] and Alger et al. [5] extended such a hypothesis and showed that flowers serve as a hub for transmitting honey bee diseases to other pollinators, suggesting that the circulation of honey bee viruses in the pollinator community may be more common than previously thought [14,15]. Indeed, other minor or non-pollinator insect species—such as ants—were also reported to test positive for several honey bee viruses [7,16,17,18]. This provides additional support to the hypothesis of interspecific associations likely serving as a conduit for virus transmission, given that ants are frequently found to interact with honey bees via activities such as raiding honey bee broods, scavenging honey bees and competing on floral resources [7,9,19,20].

Several preliminary attempts have been made to empirically test whether interaction-mediated viral transmission between ants and honey bees holds, and the results appear promising. For example, Payne et al. [17] surveyed six honey bee viruses in 14 ant species. They found a significantly higher prevalence level of several honey bee viruses in ants collected in apiary sites (67%) than those in non-apiary sites (15%), suggesting distance from the apiary could serve as a potential predictor for the infection status of honey bee virus in ants. However, one exception is that the invasive fire ant (*Solenopsis invicta*) represents the only species that has tested positive for honey bee viruses in non-apiary sites [17]. This suggests that additional mechanisms may shape honey bee viruses’ infection patterns in ant colonies distributed in places remote from the apiary. One possible mechanism is that extreme ecological dominance (e.g., widespread distribution and high density of foraging workers) of invasive ants in the field [2] may allow ant–honey bee interactions to be sufficiently frequent/intensive for successful viral transmission. If this is true, invasive ants may also act as a donor of honey bee viruses to other interacting arthropods.

A fundamental step towards a better understanding of invasive ants’ impact as a source of virus spillover is to understand the current status (e.g., prevalence, geographical distribution, replication, or genetic diversity) of honey bee viruses in invasive ants. In the present study, we used DWV, one of the major drivers of honey bee declines, as a model system and examined its distribution, prevalence, and the presence of intermediate form (as indicative of active replication of virus) in two widespread invasive ant species, namely the longhorn crazy ant (*Paratrechina longicornis*) and yellow crazy ant (*Anoplolepis gracilipes*), at a large geographical scale with a particular emphasis on the Asia–Pacific region. Phylogenetic analyses also were carried out to compare sequences of DWV recovered from the two invasive ant species and local honey bee populations (either obtained from GenBank or generated in this study). We attempted to reconstruct the honey bee virus’s history in invasive ants (e.g., whether invasive ants pick up a given honey bee virus locally after invasion). Our study presents the first dataset of distribution, prevalence, and demographic history of a significant honey bee virus across a large portion of the two ants’ current distribution. We provide key information to understand the potential transmission route of DWV and evaluate whether invasive ants could serve as practical source of virus spillover.

## 2. Materials and Methods

### 2.1. Sample Collection

**Figure 1:**
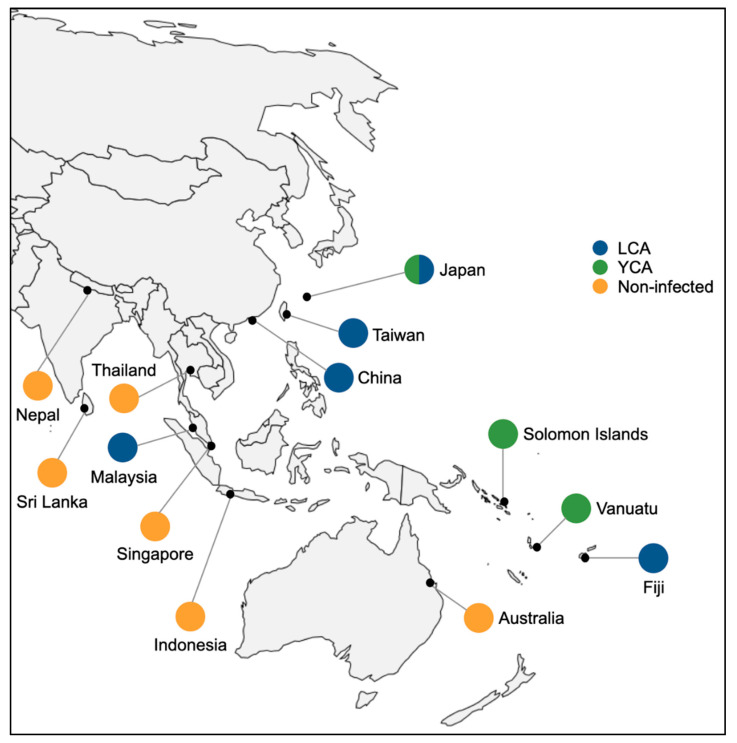
Map showing an overview of deformed wing virus status in two widespread invasive ant species, *Paratrechina longicornis* (longhorn crazy ant, LCA) and *Anoplolepis gracilipes* (yellow crazy ant, YCA), across the Asia–Pacific region.

A total of 170 and 180 colonies of longhorn crazy ant (LCA) and yellow crazy ant (YCA) were collected, respectively. The sample collection was made from 13 geographical regions across the Asia–Pacific region from 2012 to 2019 (Figure 1). All colonies were collected from sites with no apiary nearby (at least 3–5 km from any known apiary) as we aimed to understand DWV prevalence in the two ant species without interference by geographical proximity to the apiary [17]. Nine DWV-positive honey bee (*Apis mellifera*) colonies were also included as “background” sequence references (see below) and served as a positive control in subsequent molecular detections of DWV. The profile information of each sample (e.g., location and coordinates) is detailed in Table S1. Due to varying collection efforts across the regions, the sampled regions were separated into two categories, namely, “Regions with abundant collections” and “Regions with few collections” (Table 1). Data for DWV infection in local honey bee populations in each region were obtained from previously published records [21,22,23,24,25,26,27,28,29,30,31,32,33,34,35,36], Table 1.

### 2.2. RNA Isolation and RT-PCR Detection of DWV

Total RNA of five ant workers per colony of each ant species (and one honey bee worker from each of the nine colonies collected from Taiwan, Table S1) was extracted individually using TRIzol Reagent (Invitrogen, Carlsbad, CA, USA) according to the manufacturer’s protocol. RNA samples of workers from the same ant colony were mixed in the ratio of 1:1:1:1:1 and stored at −80 °C for further analysis. All mixed RNA samples (and individually-extracted RNA samples of the honey bees) were screened for the presence of DWV by virus-specific reverse transcription-polymerase chain reaction (RT-PCR), using a primer pair that amplifies partial sequences in the structural polyprotein region (DWV-F (5′-CTTACTCTGCCGTCGCCCA-3′) and DWV-R (5′-CCGTTAGGAACTCATTATCGCG-3′)) with the same protocol, PCR conditions and thermal cycling profile as described in Chen et al. [37]. DWV presence was confirmed if a specific band with the expected size (194 bp) was yielded during gel electrophoresis. As the primer pair was appliable for detecting both DWV-A and DWV-B genotypes [37], false negatives as a result of genotype-specific amplification were not expected. Proper positive and negative controls (DWV-infected and DWV-free honey bee worker samples, respectively) were included in each batch of RT-PCR reaction. Prevalence was estimated by dividing the number of DWV-positive colonies over the total number of tested colonies for both ant species in each region. We also included prevalence data from multiple previous studies to show DWV status in local honey bee populations in our sampled geographical regions [21,22,23,24,25,26,27,28,29,30,31,32,33,34,35,36], Table 1. To confirm whether the viruses are actively replicating in the two invasive ant species, a modified RT-PCR was used to detect the RNA negative strands of DWV. cDNA synthesis (PrimeScript^TM^ 1st strand cDNA Synthesis Kit, Takara Bio Inc., Shiga, Japan) was performed with a tagged forward primer (DWV Tag-F (5′-TCATGGTGGCGAATAACTTACTCTGCCGTCGCCCA-3′)). Tag primer and the corresponding reverse primer (DWV-R (5′-CCGTTAGGAACTCATTATCGCG-3′)) were used for PCR reaction. The PCR amplification protocol included 94 °C for 3 min, then 35 cycles of 94 °C for 30 s, 60 °C for 30 s, and 72 °C for 30 s, with a final extension step of 72 °C for 5 min. The PCR amplicons were confirmed by both gel electrophoresis and then sent to Sanger sequencing.

### 2.3. Phylogenetic Analysis

**Figure 2:**
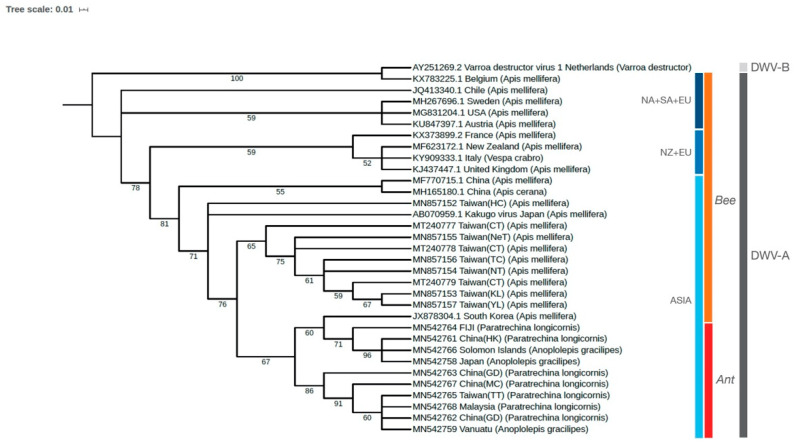
Maximum-likelihood phylogenetic analysis of multiple DWV isolates worldwide, based on the *Peptidase C3G* gene’s nucleotide sequence. The number at each branch of the phylogenetic tree represents the bootstrap value (5000 replicates). NA: North America; SA: South America; EU: Europe; NZ: New Zealand.

A separate primer pair, namely DWV-7510F (5′-ACAAGTAGACGCTGCTGTGA-3′) and DWV-8148R (5′-CCAAAGCCATGCAATCCTTC-3′) was employed to amplify the partial sequence of the *Peptidase C3G* (639 bp) for phylogenetic analysis. These primers were selected because most previously published primers, including those used for viral phylogeny, consistently yielded unspecific amplification (e.g., amplification of partial ant genomes). Blasting the two primers with all available ant genomes in the Hymenoptera Genome Database [38] resulted in the absence of predicted amplification, further verifying specificity of the two primers. The cDNA was prepared from an additional worker from ant colonies previously confirmed to be positive for DWV (Section 2.2) by PrimeScript^TM^ 1st strand cDNA Synthesis Kit (Takara Bio Inc., Shiga, Japan) according to the manufacturer’s instructions, and used as a template for subsequent PCR amplification, following similar RT-PCR conditions and cycling profile as reported in Hsu et al. [39]. Amplicons were confirmed by electrophoresis visualized on a UV transilluminator. All *Peptidase C3G* PCR products with predicted sizes were sent to Sanger sequencing at Eurofins Genomics (Tokyo, Japan). The nucleotide sequences were then compared against known DWV viral sequences on GenBank by BLAST to confirm viral identity. Viral sequences of DWV from ants and honey bees were deposited in GenBank (Accession numbers: MN542758-MN542759, MN542761-MN542768, MN857152-MN857157 and MT240777-MT240779, Table S2). The generated sequences of *Peptidase C3G* gene recovered from all collected samples in this study (including ants and honey bees), along with others obtained from GenBank (see Figure 2 for more details), were aligned using MUSCLE [40]. The phylogenetic tree based on the maximum likelihood method was reconstructed using RAxML-NG [41] with 5000 bootstrap replicates. The weighted Robinson–Foulds (WRF) distance was calculated, and bootstrapping convergence was considered to be reached if more than 99% permutations have low WRF distances, which is <3%. The generated phylogenetic tree was visualized using iTOL v5 [42]. The best evolutionary model selection was performed using ModelTest-NG (v0.1.6) [43], and GTR + I + G4 model was selected based on the Akaike information criterion (AICc). For visualization, we re-rooted tree at the clade of DWV from Belgium (host: *Apis mellifera*; KX783225.1) and Varroa destructor virus from the Netherlands (host: *Varroa destructor*; AY251269.2)**.**

## 3. Results

### 3.1. DWV Prevalence in LCA and YCA

The deformed wing virus was detected in both LCA and YCA. The overall DWV prevalence seemed slightly higher in LCA than in YCA across the sampled regions (8.8% vs. 1.67%, Table 1). In regions with a large number of collections (hereinafter referred to as “abundant collections”), the LCA’s viral prevalence ranged from 4.6% to 15.4%. In contrast, only 0.6% of YCA colonies (one out of 146) were detected as being positive for DWV (Table 1). In regions with few collections (hereinafter referred to as ‘’few collections”), the percentage of colonies that tested positive for the virus varied substantially for both ant species, across regions, and this was most likely due to sampling bias as the number of screened colonies was generally small. Our literature survey indicated that DWV was reported in local honey bee populations of all “abundant collection” regions, except Malaysia, as well as in four “few collection” regions (China, Nepal, Sri Lanka, and Thailand). The comparison of DWV status between the ants and respective local honey bees was focused only on “abundant collection” regions to avoid potential bias due to insufficient sampling. The DWV status in the sampled LCA and their respective local honey bee populations corroborated each other, but not the YCA. Deformed wing virus was detected in LCA collected in Japan, Taiwan, China and Malaysia, where local honey bees were reported with DWV infection. Interestingly, among those regions with no available information on DWV infection in local honey bee populations, DWV-positive colonies were detected in at least one ant species (Malaysia, Solomon Islands, Vanuatu, and Fiji). Negative-strand DWV was detected in all DWV-positive honey bee samples and a single DWV-positive LCA colony in Taiwan. Nevertheless, no evidence of viral replication was found in DWV-positive colonies of YCA.

### 3.2. Phylogenetic Relationships of DWV in Ants and Honey Bees

Phylogenetic analysis showed that all DWV sequences recovered from the ants (LCA: seven sequences; YCA: three sequences) were closely related to each other (belonging to the DWV-A genotype) and formed a major clade that can be further divided into two subclades. One of the subclades was closely related to DWV isolated from honey bees in South Korea (Figure 2). DWV sequences from honey bees in Taiwan formed a group except one isolate (sample name: “Taiwan(HC)”, Figure 2) and were related to both Kakugo virus isolates from Japan and the major clade comprising all ant-associated DWV (Figure 2). Overall, it is evident that sequences of all ant-associated DWV recovered from this study were most genetically similar to those isolates from honey bees in Asia.

## 4. Discussion

### 4.1. DWV Prevalence in Globally Invasive Ants

While the finding of honey bee virus-infected ants is not novel, most previous studies of such kind have either been conducted at a local scale or have focused on a single ant species. Our attempt to characterize such a pattern in invasive ants over a broad geographical range provides the first dataset that may reflect common factors shaping honey bee virus status in ants. First, the present study demonstrates that DWV persists in the longhorn crazy ant in all “abundant collection” regions, yet the level of prevalence is generally low. As all colonies of this ant were collected from sites geographically distant from any known apiary, such a low level of prevalence is expected and consistent with previous studies concerning invasive ant species (red imported fire ant [17] and Argentine ant [18]). Payne et al. [17] found that DWV is four to five times less prevalent in ants collected from non-apiary sites than those from apiary sites. Dobelmann et al. [18] reported that all ant samples that tested positive for DWV were collected only from apiary sites. All these data imply that geographic proximity of the ant colony to an apiary represents a pivotal factor in determining DWV status in ants (e.g., presence/absence and prevalence level) and raises a critical question of how DWV arriving in ant colonies is distributed in non-apiary sites. Payne et al. [17] suspect that the presence of DWV may result from ant’s consumption of carcasses of the virus-infected honey bees at distances away from the colony. Viral transmission via scavenging honey bees may apply in the longhorn crazy ant as this ant species is well-known for its long-distance foraging from the colony [44]. Two more potential mechanisms exist: (1) human-assisted long-distance spread is relatively common in invasive ants [45], with the longhorn crazy ant being no exception. DWV-positive ant colonies inhabiting in or nearby an apiary could be jump dispersed to somewhere distant from the apiary by the assistance of human transportation; (2) many invasive ant species form a unique social structure termed a “supercolony” in which physically separated nests are mutually tolerant to each other but aggressive to those belonging to a different supercolony. Such population structure has been shown to facilitate the horizontal transmission of pathogens among nests of invasive ants in an extensively large area due to the lack of visible nest boundaries that allows inter-nest interactions [39]. It would be interesting to test if DWV can be transmitted horizontally among ant nests within the same supercolony, especially given that DWV (and other honey bee viruses) is generally present at a low titer within ants [46,47].

One notable finding is that the presence of DWV is sporadic in the yellow crazy ant. Sampling bias can be excluded as nearly equal survey effort between the two ant species was ensured. Our preliminary data also show that virtually all colonies of yellow crazy ant collected in apiary-sites were negative for DWV infection (Lin et al., unpublished data). These data, coupled with a similar pattern in another population (Australia) of this ant by Cooling et al. [48], suggest that the window for successful interspecific transmission of DWV is likely narrow in the yellow crazy ant. Although speculative, additional factors such as the immune responses of ants may have been involved [46]. For example, we argue that competition between coinfecting pathogens may partially explain DWV’s low incidence in the yellow crazy ant. Most of our sampled regions are located in the putative native range of the yellow crazy ant (e.g., Southeast Asia) [49,50], where diversity of naturally-infecting pathogens, including viruses, is presumably higher than in introduced populations [51,52]. This means that pathogen–pathogen competition, especially between those sharing a similar niche [53], may have been more intense in the native host population, thus leaving limited resources for the persistence of non-specific viruses, such as honey bee viruses. At least two lines of evidence are supportive of this possibility: (1) all DWV-positive colonies of yellow crazy ant are only found in the known introduced range of this ant (Table 1); (2) we have identified at least eight single-strain RNA virus species (Table S3) in the yellow crazy ant across the Asia–Pacific regions, and most of which were either undetected or at relatively low prevalence in populations outside the putative native range ([54], Lee et al. under review), suggesting that pathogen–pathogen competition may have been mostly relaxed and thus a higher likelihood for the survival of DWV could be ensured once being transmitted to the ant.

### 4.2. Origin of DWV in Invasive Ants

One explanation for DWV’s presence in the invasive ants is that DWV may arrive as a hitch-hiker with the ant species during an introduction event (e.g., the introduction of ant colonies that carry the virus) and somewhat persist in the introduced range of the ant. Our data, however, provide little support to this notion and remain in favor of local interspecific transmission after the introduction of the ant. First, our survey reveals that DWV-positive colonies of the longhorn crazy ants can be generally found in regions where DWV-infected honey bees were also reported (Table 1), suggesting that interspecific transmission of DWV from the honey bees to the ants is possible. Second, Australia is one of the very few locations in which European honey bees are reported to be free of DWV [34,55,56], and DWV is absent in several invasive ant species established in Australia, including the two crazy ant species in this study, as well as the Argentine ant [7,16,52]. Third, our phylogenetic analyses reveal a grouping of DWV sequences by their geographical regions (i.e., Asian group vs. European + New Zealand group vs. European+ North and South American group). We found that most of the DWV sequences recovered from the two ant species are genetically similar to those Asian isolates in honey bees (i.e., China, Korea, Japan, and Taiwan). These findings corroborate with Dobelmann et al. [18] and further support the prediction of post-invasion acquisition of DWV by ants from the local environment and/or other host species coinhabiting the same location. An exception occurs when analyzing DWV in ants collected from the Pacific Islands (i.e., Solomon Islands, Fiji, and Vanuatu). Given the fact that no naturally occurring honey bees exist on these islands [57] and that European honey bee populations there are mostly descendants of bees imported from Australia and New Zealand [58], one should expect an infection status of DWV in honey bee mostly resembling that in one of the two import origins (e.g., DWV-free honey bee population or infection by isolates genetically similar to those in New Zealand). However, DWV sequences in ants are invariably clustered together with Asian isolates, suggesting that DWV in ants in these islands may not originate from local honey bees. A more complex origin may be involved. Previous and ongoing monitoring effort shows no evidence for DWV infections in honey bees in the three Pacific islands [32,33,35,36,59], hence, detection of the presence of DWV and examination of its sequence diversity in a diverse range of local arthropod hosts is needed. These data would serve as baseline information to understand DWV’s origin and the dynamics of DWV transmission among interacting arthropods in an island ecosystem.

### 4.3. The role of Invasive Ants in DWV Transmission 

DWV has been detected in more than 60 insect species (including two ant species) that span eight orders [56]. Another recent study even extended the number of DWV-positive ant species to 12 [60], suggesting this virus may have a rather wide host range [61] and that ants are likely a natural host of DWV. While true infection of DWV (as evident by the presence of the replicative form of the virus) is only found in a few ant species (including the longhorn crazy ant in this study) [7,16,18,60,62], this does not necessarily preclude ants as a potential DWV host, but may simply reflect the lack of systematic survey effort on the status of viral infection in these ant species. Future research with a systematic, multi-scale design (e.g., different geographical populations, different castes/life stages of ants or even testing the efficiency of foodborne virus transmission [47,62]) is therefore needed in order to close the given knowledge gaps. 

While it remains ambiguous that the two invasive ants in this study are natural hosts of DWV, our findings in this study may help evaluate whether invasive ants would serve as a transmission source of DWV from at least two fundamental perspectives. First, DWV prevalence in both invasive ants is generally low within the area that they are distributed, unless colonies are physically located in or near an apiary where interactions between honey bees and ants are sufficiently intense to allow frequent virus transfer from bees to ants [17]. This implies that the widespread distribution of invasive ants in their introduced environments may not necessarily translate to increased exposure of recipient hosts to DWV (e.g., higher probability of encountering an infected host), as the accumulation of high viral levels are primarily centered in apiaries or colonies nearby. Second, viral replication is found in one species (yet is rare) but not the other in this study, indicating that propagation of DWV in the two invasive ant species may not be efficient and thus, that DWV transmission mediated by these ants is likely scarce in the field.

## Figures and Tables

**Table 1:** Prevalence of deformed wing virus (DWV) in two invasive ant species, *Paratrechina longicornis* (longhorn crazy ant, LCA) and *Anoplolepis gracilipes* (yellow crazy ant, YCA), across the Asia–Pacific region.

Region	DWV		
	LCA	YCA	Reference
	Prevalence (%) (No. of DWV—positive colony/total no. of the tested colony) [^a^]		(+/−) [^b^]
Regions with Abundant Collections ^c^			
Japan	15.4 (2/13)	3.4 (1/29)	+ [21,22]
Taiwan	4.6 (4/87)	− (0/68)	+ [23,24]
Malaysia	13.6 (3/22)	− (0/49)	N ^d^
Regions with Few Collections ^c^			
China	26.7 (4/15)	− (0/4)	+ [25,26]
Nepal	− (0/4)		+ [27]
Indonesia	− (0/2)	− (0/5)	− [28]
Sri Lanka		− (0/1)	+ [27]
Thailand	− (0/9)	− (0/7)	+ [29,30,31]
Singapore	− (0/5)	− (0/11)	N ^d^
Solomon Island		100 (1/1)	− [32,33]
Australia	− (0/7)	− (0/2)	− [34]
Vanuatu		50 (1/2)	− [35]
Fiji	33.3 (2/6)	− (0/1)	− [36]
Total	8.82% (170)	1.67% (180)	

^a^ Blank: no samples collected; ^b^ References; +: detected; −: non-detected. ^c^ Refer to Table S1 for more details; ^d^ No reference available.

## Supplementary Materials

The following are available online at https://www.mdpi.com/1999-4915/12/11/1309/s1, Table S1: Geographic coordinates and locations of all surveyed invasive ants and honey bees in this study, Table S2: GenBank accession numbers of DWV isolated in this study, Table S3: Single-strand RNA viruses (published/under review/unpublished) discovered in the yellow crazy ant, *Anoplolepis gracilipes* (Note that all the viruses are found widespread in the ant’s putative native range, but persist at low prevalence or are absent in most of the ant’s known introduced ranges such as East Asia).
