# Supplementary material for: Deformed Wing Virus in Two Widespread Invasive Ants: Geographical Distribution, Prevalence, and Phylogeny
Source: Viruses. 2020 Nov 15;12(11):1309. doi: 10.3390/v12111309 (PMC7696054; doi:10.3390/v12111309)
Supplement: Supplementary file 1 [file viruses-12-01309-s001.pdf]

Table S1. Geographic coordinates and locations of all surveyed invasive ants and honey bees in this study

Sample No.	Sample name	Coordinate	Location
1	PL-TW1	25.1574, 121.4016	Taipei, Taiwan
2	PL-TW2	25.0563, 121.2246	Taoyuan, Taiwan
3	PL-TW3	24.8280, 121.0712	Hsinchu, Taiwan
4	PL-TW4	24.8184, 121.1281	Hsinchu, Taiwan
5	PL-TW6	24.4951, 120.8273	Miaoli, Taiwan
6	PL-TW12	24.0826, 120.5584	Changhua, Taiwan
7	PL-TW17	23.7580, 120.6715	Nantou, Taiwan
8	PL-TW25	23.1365, 120.3002	Tainan, Taiwan
9	PL-TW29	22.6557, 120.2913	Kaohsiung, Taiwan
10	PL-TW33	24.9022, 121.8620	Yilan, Taiwan
11	PL-TW39	23.7712, 120.4127	Yunlin, Taiwan
12	PL-TW47	23.4113, 120.3081	Chiayi, Taiwan
13	PL-TW55	22.4585, 120.4798	Pintung, Taiwan
14	PL-TW60	22.7133, 120.5292	Pintung, Taiwan
15	PL-TW61	22.6982, 120.3482	Kaohsiung, Taiwan
16	PL-TW62	22.9259, 120.3444	Tainan, Taiwan
17	PL-TW64	23.6089, 120.5452	Yunlin, Taiwan
18	PL-TW66	24.9431, 121.1554	Taoyuan, Taiwan
19	PL-TW70	23.8946, 121.5487	Hualien, Taiwan
20	PL-TW75	24.0083, 121.6146	Hualien, Taiwan
21	PL-TW81	23.4979, 121.3775	Hualien, Taiwan
22	PL-TW83	23.5647, 121.3819	Hualien, Taiwan
23	PL-TW87	24.3381, 120.7738	Miaoli, Taiwan
24	PL-TW91	24.2703, 120.5575	Taichung, Taiwan

25	PL-TW92	24.3112, 120.5502	Taichung, Taiwan
26	PL-TW94	23.5712, 120.3351	Chiayi, Taiwan
27	PL-TW96	24.1683, 120.6396	Taichung, Taiwan
28	PL-TW99	24.1025, 120.6756	Taichung, Taiwan
29	PL-TW101	24.1041, 120.6782	Taichung, Taiwan
30	PL-TW103	23.8872, 120.4709	Changhua, Taiwan
31	PL-TW108	24.1187, 120.6718	Taichung, Taiwan
32	PL-TW112	25.0173, 121.5407	Taipei, Taiwan
33	PL-TW121	24.1279, 120.6849	Taichung, Taiwan
34	PL-TW131	24.2025, 120.7280	Taichung, Taiwan
35	PL-TW134	23.8266, 120.7887	Nantou, Taiwan
36	PL-TW139	24.1410, 120.6456	Taichung, Taiwan
37	PL-TW144	22.6024, 120.5610	Pintung, Taiwan
38	PL-TW146	22.6698, 120.5039	Pintung, Taiwan
39	PL-TW152	22.6101, 120.5663	Pintung, Taiwan
40	PL-TW154	22.6590, 120.5589	Pintung, Taiwan
41	PL-TW157	22.5737, 120.6101	Pintung, Taiwan
42	PL-TW160	22.5077, 120.5879	Pintung, Taiwan
43	PL-TW163	22.5440, 120.4615	Pintung, Taiwan
44	PL-TW166	22.6121, 120.2999	Kaohsiung, Taiwan
45	PL-TW167	22.5882, 120.3225	Kaohsiung, Taiwan
46	PL-TW169	22.6270, 120.3014	Kaohsiung, Taiwan
47	PL-TW172	22.6257, 120.3637	Kaohsiung, Taiwan
48	PL-TW174	25.0205, 121.3697	Taoyuan, Taiwan
49	PL-TW176	23.0033, 120.2096	Tainan, Taiwan
50	PL-TW179	22.9977, 120.2094	Tainan, Taiwan
51	PL-TW180	22.7578, 121.1027	Taitung, Taiwan
52	PL-TW181	22.8537, 121.1040	Taitung, Taiwan

53	PL-TW182	22.7754, 121.1476	Taitung, Taiwan
54	PL-TW185	23.5671, 119.5651	Penghu, Taiwan
55	PL-TW186	23.5675, 119.5620	Penghu, Taiwan
56	PL-TW187	23.5560, 119.6020	Penghu, Taiwan
57	PL-TW189	23.6605, 119.5600	Penghu, Taiwan
58	PL-TW190	23.6324, 119.5136	Penghu, Taiwan
59	PL-TW195	22.0637, 121.5661	Orchid island, Taiwan
60	PL-TW200	23.5636, 119.4894	Penghu, Taiwan
61	PL-TW202	23.5641, 119.4744	Penghu, Taiwan
62	PL-TW214	23.8889, 120.5396	Changhua, Taiwan
63	PL-TW215	24.7035, 121.1832	Hsinchu, Taiwan
64	PL-TW217	24.6980, 121.1891	Hsinchu, Taiwan
65	PL-TW219	24.5205, 121.8330	Yilan, Taiwan
66	PL-TW221	23.3676, 121.3266	Hualien, Taiwan
67	PL-TW225	22.9197, 121.1397	Taitung, Taiwan
68	PL-TW228	22.3413, 120.8947	Taitung, Taiwan
69	PL-TW232	21.9952, 120.7463	Pintung, Taiwan
70	PL-TW234	22.0703, 120.7105	Pintung, Taiwan
71	PL-TW238	23.8430, 120.4810	Changhua, Taiwan
72	PL-TW239	22.08, 121.5281	Orchid island, Taiwan
73	PL-TW242	22.0331, 121.5764	Orchid island, Taiwan
74	PL-TW243	22.0382, 121.567	Orchid island, Taiwan
75	PL-TW244	22.0617, 121.5726	Orchid island, Taiwan
76	PL-TW246	25.0715, 121.5616	Taipei, Taiwan
77	PL-TW247	24.9916, 121.5435	Taipei, Taiwan
78	PL-TW251	24.1122, 120.6161	Taichung, Taiwan
79	PL-TW256	24.5962, 121.0441	Miaoli, Taiwan
80	PL-TW263	24.2084, 120.5969	Taichung, Taiwan

81	PL-TW268	24.0893, 121.0345	Nantou, Taiwan
82	PL-TW270	24.0915, 121.0331	Nantou, Taiwan
83	PL-TW297	22.6717, 121.4713	Green island, Taiwan
84	PL-TW298	22.6731, 121.4726	Green island, Taiwan
85	PL-TW312	24.4314, 118.3145	Kinmen, Taiwan
86	PL-TW313	24.3905, 118.3206	Kinmen, Taiwan
87	PL-TW314	24.4249, 118.3190	Kinmen, Taiwan
88	PL-CN2	22.2969, 114.1742	Hong Kong, China
89	PL-CN3	22.2838, 114.1586	Hong Kong, China
90	PL-CN04.2	22.2566, 113.9027	Hong Kong, China
91	PL-CN05	22.2900, 113.9390	Hong Kong, China
92	PL-CN07	22.2794, 114.1579	Hong Kong, China
93	PL-CN08	22.2870, 114.2105	Hong Kong, China
94	PL-CN09	22.2639, 114.2371	Hong Kong, China
95	PL-CN11	22.1961, 113.5411	Macau, China
96	PL-CN12	22.5021, 114.1265	Hong Kong, China
97	PL-CN13	22.4130, 114.2101	Hong Kong, China
98	PL-CN16	22.5370, 114.0540	Guangdong, China
99	PL-CN17	22.5466, 114.1267	Guangdong, China
100	PL-CN19	23.3617, 116.0348	Guangdong, China
101	PL-CN20	23.2968, 116.1711	Guangdong, China
102	PL-CN21	23.4211, 116.0490	Guangdong, China
103	PL-JP01	26.2189, 127.6864	Okinawa, Japan
104	PL-JP02	26.1784, 127.7994	Okinawa, Japan
105	PL-JP04	24.3903, 124.2469	Okinawa, Japan
106	PL-JP6	24.3310, 123.9090	Okinawa, Japan
107	PL-JP07	26.3987, 127.7580	Okinawa, Japan
108	PL-JP08	26.3335, 127.7870	Okinawa, Japan

109	PL-JP9	26.1673, 127.8288	Okinawa, Japan
110	PL-JP10	26.1447, 127.6647	Okinawa, Japan
111	PL-JP11	26.0957, 127.6827	Okinawa, Japan
112	PL-JP13	26.1418, 127.7489	Okinawa, Japan
113	PL-JP14	26.1377, 127.7290	Okinawa, Japan
114	PL-JP15	26.6776, 127.8912	Okinawa, Japan
115	PL-JP21	26.4361, 127.7930	Okinawa, Japan
116	PL-MY49	5.4019, 100.2761	Penang, Malaysia
117	PL-MY51	5.4075, 100.2782	Penang, Malaysia
118	PL-MY52	5.3443, 100.3016	Penang, Malaysia
119	PL-MY53	5.3018, 100.2756	Penang, Malaysia
120	PL-MY54	5.3050, 100.2691	Penang, Malaysia
121	PL-MY55	5.2862, 100.2328	Penang, Malaysia
122	PL-MY56	5.2822, 100.2173	Penang, Malaysia
123	PL-MY57	5.3260, 100.2207	Penang, Malaysia
124	PL-MY58	5.3265, 100.2057	Penang, Malaysia
125	PL-MY59	5.3583, 100.2251	Penang, Malaysia
126	PL-MY63	5.4185, 100.3325	Penang, Malaysia
127	PL-MY64	5.4120, 100.3404	Penang, Malaysia
128	PL-MY65	5.4381, 100.2898	Penang, Malaysia
129	PL-MY70	5.3504, 100.4460	Penang, Malaysia
130	PL-MY71	5.3557, 100.2961	Penang, Malaysia
131	PL-MY73	5.4382, 102.2093	Kelantan, Malaysia
132	PL-MY75	5.3532, 100.2960	Penang, Malaysia
133	PL-MY101	3.4217, 115.1526	Sarawak, Malaysia
134	PL-MY102	1.5575, 110.3479	Sarawak, Malaysia
135	PL-MY105	5.3572, 100.3033	Penang, Malaysia
136	PL-MY109	5.3369, 100.2938	Penang, Malaysia

137	PL-MY136	5.3949, 100.3108	Penang, Malaysia
138	PL-TH01	13.8421, 100.5729	Bangkok, Thailand
139	PL-TH11	13.8028, 100.5533	Bangkok, Thailand
140	PL-TH13	13.8062, 100.5551	Bangkok, Thailand
141	PL-TH14	13.8081, 100.5567	Bangkok, Thailand
142	PL-TH20	14.4579, 100.5377	Phra Nakhon Si Ayutthaya, Thailand
143	PL-TH28	14.3501, 100.5423	Phra Nakhon Si Ayutthaya, Thailand
144	PL-TH31	13.6800, 100.6601	Bangkok, Thailand
145	PL-TH32	14.2281, 100.7068	Phra Nakhon Si Ayutthaya, Thailand
146	PL-TH39	14.5146, 101.9591	Nakhon Ratchasima, Thailand
147	PL-SG30	1.3123, 103.9396	Singapore
148	PL-SG31	1.3353, 103.7455	Singapore
149	PL-SG32	1.2940, 103.8539	Singapore
150	PL-SG33	1.3380, 103.7061	Singapore
151	PL-SG34	1.3385, 103.7431	Singapore
152	PL-AU5	-16.9280, 145.7787	Cairns, Australia
153	PL-AU7	-16.9260, 145.7747	Cairns, Australia
154	PL-AU8	-16.9229, 145.7708	Cairns, Australia
155	PL-AU10	-16.9299, 145.7768	Cairns, Australia
156	PL-AU20	-16.8736, 145.7555	Cairns, Australia
157	PL-AU25	-16.9268, 145.7780	Cairns, Australia
158	PL-FJ1	-17.7728, 177.3671	Denarau island, Fiji
159	PL-FJ2	-18.1058, 178.3953	Suva, Fiji
160	PL-FJ4	-18.1458, 178.4475	Suva, Fiji
161	PL-FJ6	-17.4491, 177.9830	Viti Levu, Fiji

162	PL-FJ8	-18.2100, 177.7116	Queens Rd., Fiji
163	PL-FJ9	-18.0880, 177.5522	Sigatoka, Fiji
164	PL-ID KT	-0.8280, 100.5302	Sumatera Barat, Indonesia
165	PL-ID DROM	-6.2286, 106.8185	Jakarta, Indonesia
166	PL-07.561	16.79, -62.211	Brades
167	PL-NP11	27.7144, 85.2928	Swayambhu, Nepal
168	PL-NP13	28.1900, 83.9589	Pokhara, Nepal
169	PL-NP15	28.2217, 83.9546	Pokhara, Nepal
170	PL-NP17	28.1946, 83.9693	Pokhara, Nepal
171	Ano-TW01	24.4949, 120.8277	Miaoli, Taiwan
172	Ano-TW02	22.4583, 120.4806	Pintung, Taiwan
173	Ano-TW03	24.9358, 121.1880	Taoyuan, Taiwan
174	Ano-TW05	23.899, 121.5502	Hualien, Taiwan
175	Ano-TW6	23.8985, 121.5357	Hualien, Taiwan
176	Ano-TW09	24.8244, 121.0819	Hsinchu, Taiwan
177	Ano-TW10	23.4856, 120.4682	Chiayi, Taiwan
178	Ano-TW11	24.8253, 121.0864	Hsinchu, Taiwan
179	Ano-TW12	24.9072, 121.8500	Yilan, Taiwan
180	Ano-TW17	24.1364, 120.6851	Taichung, Taiwan
181	Ano-TW18	24.1242, 120.6561	Taichung, Taiwan
182	Ano-TW19	22.8949, 120.6223	Kaohsiung, Taiwan
183	Ano-TW20	25.0205, 121.3697	Taoyuan, Taiwan
184	Ano-TW21	25.1203, 121.8596	Taipei, Taiwan
185	Ano-TW24	24.2265, 120.5783	Taichung, Taiwan
186	Ano-TW25	22.5311, 120.9633	Taitung, Taiwan
187	Ano-TW27	24.9276, 121.2805	Taoyuan, Taiwan
188	Ano-TW28	25.0872, 121.6227	Taipei, Taiwan
189	Ano-TW30	24.6001, 121.0399	Miaoli, Taiwan

190	AG-TW031	21.9248, 120.8345	Pintung, Taiwan
191	AG-TW032	21.9241, 120.8340	Pintung, Taiwan
192	AG-TW033	21.9252, 120.8345	Pintung, Taiwan
193	AG-TW034	21.9252, 120.8346	Pintung, Taiwan
194	AG-TW035	21.9251, 120.8343	Pintung, Taiwan
195	AG-TW036	21.9246, 120.8346	Pintung, Taiwan
196	AG-TW037	21.9248, 120.8346	Pintung, Taiwan
197	AG-TW038	21.9246, 120.8342	Pintung, Taiwan
198	AG-TW039	21.9249, 120.8341	Pintung, Taiwan
199	Ano-TW39	24.6973, 120.8575	Miaoli, Taiwan
200	AG-TW040	21.925, 120.8342	Pintung, Taiwan
201	AG-TW041	21.9237, 120.8337	Pintung, Taiwan
202	AG-TW042	21.9238, 120.8339	Pintung, Taiwan
203	AG-TW043	21.9907, 120.8553	Pintung, Taiwan
204	Ano-TW43	24.6825, 121.3796	Taoyuan, Taiwan
205	AG-TW044	21.9270, 120.8298	Pintung, Taiwan
206	AG-TW045	21.9271, 120.8299	Pintung, Taiwan
207	AG-TW046	21.9272, 120.8299	Pintung, Taiwan
208	AG-TW047	23.4713, 120.4841	Chiayi, Taiwan
209	AG-TW048	23.4709, 120.4841	Chiayi, Taiwan
210	AG-TW049	23.4733, 120.4856	Chiayi, Taiwan
211	AG-TW50	23.4716, 120.4893	Chiayi, Taiwan
212	AG-TW051	24.8298, 121.0832	Hsinchu, Taiwan
213	AG-TW052	24.8205, 121.0971	Hsinchu, Taiwan
214	AG-TW053	24.8506, 121.0809	Hsinchu, Taiwan
215	AG-TW054	25.1907, 121.4279	New Taipei, Taiwan
216	AG-TW055	25.1752, 121.4316	New Taipei, Taiwan
217	AG-TW056	25.1519, 121.4908	Taipei, Taiwan

218	AG-TW057	22.7065, 121.0259	Taitung, Taiwan
219	Ano-TW60	22.9984, 120.2186	Taitung, Taiwan
220	Ano-TW63	23.9121, 120.8870	Nantou, Taiwan
221	Ano-TW65	24.4517, 118.3494	Kinmen, Taiwan
222	Ano-TW67	22.0842, 120.8354	Pintung, Taiwan
223	Ano-TW73	23.9311, 121.4994	Hualien, Taiwan
224	Ano-TW75	23.9354, 121.5069	Hualien, Taiwan
225	Ano-TW76	24.1791, 121.5079	Hualien, Taiwan
226	Ano-TW80	22.5291, 120.9394	Taitung, Taiwan
227	Ano-TW85	25.0150, 121.5385	Taipei, Taiwan
228	Ano-TW86	24.0624, 120.7384	Taichung, Taiwan
229	Ano-TW99	25.0882, 121.6233	Taipei, Taiwan
230	Ano-TW102	25.1546, 121.5392	Taipei, Taiwan
231	Ano-TW106	24.6102, 121.8276	Yilan, Taiwan
232	Ano-TW107	24.6129, 121.8261	Yilan, Taiwan
233	Ano-TW108	23.9658, 121.4857	Hualien, Taiwan
234	Ano-TW110	24.2998, 121.7514	Hualien, Taiwan
235	Ano-TW117	22.0143, 121.5723	Orchid island, Taiwan
236	Ano-TW118	22.6712, 121.4677	Green island, Taiwan
237	Ano-TW123	22.9013, 121.1518	Taitung, Taiwan
238	Ano-MY01	5.6618, 100.5001	Negeri Kedah, Malaysia
239	Ano-MY02	5.4400, 100.2872	Penang, Malaysia
240	AG-MY003	5.3574, 100.3025	Penang, Malaysia
241	AG-MY004	5.359, 100.3025	Penang, Malaysia
242	Ano-MY05	5.3547, 100.3007	Penang, Malaysia
243	AG-MY005	5.3589, 100.3077	Penang, Malaysia
244	AG-MY006	5.3472, 100.2954	Penang, Malaysia
245	Ano-MY07	2.2719, 102.2979	Maleka, Malaysia

246	AG-MY007	5.3431, 100.284	Penang, Malaysia
247	Ano-MY08	1.8413, 102.9552	Johor, Malaysia
248	AG-MY008	5.3427, 100.284	Penang, Malaysia
249	Ano-MY09	5.6144, 100.4839	Negeri Kedah, Malaysia
250	AG-MY009	5.3427, 100.284	Penang, Malaysia
251	AG-MY010	5.3428, 100.284	Penang, Malaysia
252	AG-MY011	5.3427, 100.284	Penang, Malaysia
253	Ano-MY11	5.3650, 100.3944	Penang, Malaysia
254	AG-MY014	5.3436, 100.2846	Penang, Malaysia
255	AG-MY015	5.3435, 100.2845	Penang, Malaysia
256	AG-MY016	5.4667, 100.2031	Penang, Malaysia
257	AG-MY017	5.4675, 100.2004	Penang, Malaysia
258	Ano-MY17	3.3415, 101.2446	Negeri Selangor, Malaysia
259	AG-MY018	5.4676, 100.2009	Penang, Malaysia
260	AG-MY019	5.4678, 100.2014	Penang, Malaysia
261	Ano-MY19	4.7729, 100.7623	Perak, Malaysia
262	AG-MY020	5.4683, 100.2029	Penang, Malaysia
263	AG-MY021	5.3498, 100.252	Penang, Malaysia
264	AG-MY022	5.349, 100.2519	Penang, Malaysia
265	Ano-MY22	5.4602, 100.2062	Penang, Malaysia
266	AG-MY023	5.3692, 100.2481	Penang, Malaysia
267	AG-MY024	5.3694, 100.2482	Penang, Malaysia
268	Ano-MY24	5.3939, 100.2634	Penang, Malaysia
269	AG-MY025	5.4134, 100.271	Penang, Malaysia
270	AG-MY026	5.4203, 100.2661	Penang, Malaysia
271	Ano-MY31	5.3428, 100.2823	Penang, Malaysia
272	Ano-MY34	3.1496, 101.6837	Kuala Lumpur, Malaysia
273	Ano-MY42	6.0924, 102.2746	Kelantan, Malaysia

274	Ano-MY45	1.829, 103.834	Johor, Malaysia
275	AG-MY076	5.3574, 100.3001	Penang, Malaysia
276	AG-MY077	5.3591, 100.2996	Penang, Malaysia
277	AG-MY078	5.3588, 100.3054	Penang, Malaysia
278	AG-MY079	5.3434, 100.2809	Penang, Malaysia
279	AG-MY080	5.3429, 100.2817	Penang, Malaysia
280	AG-MY081	5.3428, 100.2818	Penang, Malaysia
281	AG-MY082	5.3585, 100.3012	Penang, Malaysia
282	AG-MY084	5.3572, 100.3040	Penang, Malaysia
283	AG-MY085	5.3570, 100.3049	Penang, Malaysia
284	AG-MY086	5.3560, 100.3039	Penang, Malaysia
285	AG-MY087	5.3558, 100.3063	Penang, Malaysia
286	AG-MY088	5.3543, 100.3060	Penang, Malaysia
287	Ano-JP03	26.2271, 127.7164	Okinawa, Japan
288	Ano-JP06	26.7296, 128.1678	Okinawa, Japan
289	Ano-JP07	26.4687, 127.8292	Okinawa, Japan
290	Ano-JP12	26.5268, 127.9268	Okinawa, Japan
291	Ano-JP13	26.3995, 127.7580	Okinawa, Japan
292	Ano-JP17	26.3337, 127.7871	Okinawa, Japan
293	Ano-JP18	26.2856, 127.8039	Okinawa, Japan
294	Ano-JP20	26.1671, 127.8285	Okinawa, Japan
295	Ano-JP21	26.0956, 127.6827	Okinawa, Japan
296	Ano-JP24	26.1381, 127.7288	Okinawa, Japan
297	Ano-JP27	26.2398, 127.7235	Okinawa, Japan
298	Ano-JP30	26.6608, 128.1011	Okinawa, Japan
299	Ano-JP33	26.5543, 128.0402	Okinawa, Japan
300	Ano-JP39	26.6913, 127.8792	Okinawa, Japan
301	Ano-JP41	26.6045, 127.9593	Okinawa, Japan

302	Ano-JP42	26.2715, 127.7422	Okinawa, Japan
303	Ano-JP44	26.3646, 127.8535	Okinawa, Japan
304	AG-JP059	26.1494, 127.6611	Okinawa, Japan
305	AG-JP061	26.1081, 127.6696	Okinawa, Japan
306	AG-JP062	26.108, 127.6695	Okinawa, Japan
307	AG-JP063	26.1074, 127.6721	Okinawa, Japan
308	AG-JP065	26.0954, 127.6914	Okinawa, Japan
309	Ano-JP67	26.665, 128.0122	Okinawa, Japan
310	AG-JP67	26.672, 128.0114	Okinawa, Japan
311	AG-JP069	26.6742, 128.0103	Okinawa, Japan
312	AG-JP070	26.855, 128.2494	Okinawa, Japan
313	AG-JP071	26.4590, 127.8365	Okinawa, Japan
314	AG-JP072	26.4469, 127.8037	Okinawa, Japan
315	AG-JP074	26.1357, 127.7706	Okinawa, Japan
316	Ano-Okinawa	26.4373, 127.7998	Okinawa, Japan
317	Ano-CN06	22.2493, 113.5491	Guangdong, China
318	Ano-CN07	22.2425, 113.5440	Guangdong, China
319	Ano-HK1	22.2836, 114.1370	Hong Kong, China
320	Ano-HK2	22.2830, 114.1340	Hong Kong, China
321	Ano-TH01	14.3528, 100.5324	Phra Nakhon Si Ayutthaya, Thailand
322	Ano-TH02	13.6823, 100.6597	Bangkok, Thailand
323	Ano-TH3	14.5897, 101.0233	Salavan, Thailand
324	Ano-TH4	14.8345,101.5498	Nakhon Ratchasima, Thailand
325	Ano-TH5	14.5144, 101.9590	Nakhon Ratchasima, Thailand
326	Ano-TH6	14.4344, 101.8743	Nakhon Ratchasima, Thailand
327	Ano-TH07	14.3718, 101.8637	Nakhon Ratchasima, Thailand
328	Ano-SG1	1.3364, 103.7463	Singapore

329	Ano-SG02	1.3463, 103.7592	Singapore
330	Ano-SG3	1.3480, 103.7765	Singapore
331	Ano-SG4	1.3469, 103.7759	Singapore
332	Ano-SG5	1.3476, 103.7750	Singapore
333	Ano-SG06	1.3475, 103.7749	Singapore
334	Ano-SG7	1.3339, 103.7409	Singapore
335	Ano-SG8	1.267, 103.821	Singapore
336	Ano-SG09	1.296, 103.781	Singapore
337	Ano-SG10	1.296, 103.784	Singapore
338	Ano-SG11	1.307, 103.79	Singapore
339	Ano-ID01	-6.8214, 107.5560	Jawa Barat, Indonesia
340	AG-ID027	-6.4879, 106.8593	Jawa Barat, Indonesia
341	AG-ID028	-6.5004, 106.8443	Jawa Barat, Indonesia
342	AG-ID029	-6.4993, 106.8445	Jawa Barat, Indonesia
343	AG-ID030	-6.4931, 106.8463	Jawa Barat, Indonesia
344	Ano-LK01	6.9729, 79.9136	Sri Lanka
345	Ano-AU01	-17.0456, 145.7290	Queensland, Australia
346	Ano-AU02	-17.0524, 145.7296	Queensland, Australia
347	Ano-VU1	-17.7441, 168.3307	Vanuatu
348	Ano-VU03	-19.5247, 169.4987	Vanuatu
349	Ano-FJ01	-17.298, 178.217	North island, Fiji
350	Ano-Sb1	-9.4272, 160.0028	Solomon island
I	AM-TW(HC)	24.7874, 121.1782	Hsinchu, Taiwan
II	AM-TW(KL)	25.1122, 121.6887	Keelung, Taiwan
III	AM-TW(NT)	24.0048, 121.1227	Nantou, Taiwan
IV	AM-TW(NeT)	24.9339, 121.7417	New Taipei City, Taiwan
V	AM-TW(TC)	24.1199, 120.6760	Taichung, Taiwan
VI	AM-TW(YL)	24.7472, 121.7460	Yilan, Taiwan

VII	AM-TW(CT)1	24.1199, 120.6760	Taichung, Taiwan
VIII	AM-TW(CT)2	24.1199, 120.6760	Taichung, Taiwan
IX	AM-TW(CT)3	24.1199, 120.6760	Taichung, Taiwan

Table S2. GenBank accession numbers of DWV isolated in this study

Accession number	Sample name	Location	Coordinates	Species name
Ant colonies				
MN542758	Ano-JP44	Japan	26.3646, 127.8535	*Anoplolepis gracilipes*
MN542759	Ano-VU1	Vanuatu	-17.7441, 168.3307	*Anoplolepis gracilipes*
MN542761	PL-CN2	China	22.2969, 114.1742	*Paratrechina longicornis*
MN542762	PL-CN20	China	23.2968, 116.1711	*Paratrechina longicornis*
MN542763	PL-CN21	China	23.4211, 116.0490	*Paratrechina longicornis*
MN542764	PL-FJ4	Fiji	-18.1458, 178.4475	*Paratrechina longicornis*
MN542765	PL-TW219	Taiwan	24.5205, 121.8330	*Paratrechina longicornis*
MN542766	Ano-Sb1	Solomon Islands	-9.4272, 160.0028	*Anoplolepis gracilipes*
MN542767	PL-CN11	China	22.1961, 113.5411	*Paratrechina longicornis*
MN542768	PL-MY102	Malaysia	1.5575, 110.3479	*Paratrechina longicornis*
Honey bee colonies				
MN857152	AM-TW(HC)	Hsinchu, Taiwan	24.7874, 121.1782	
MN857153	AM-TW(KL)	Keelung, Taiwan	25.1122, 121.6887	
MN857154	AM-TW(NT)	Nantou, Taiwan	24.0048, 121.1227	
MN857155	AM-TW(NeT)	New Taipei City, Taiwan	24.9339, 121.7417	
MN857156	AM-TW(TC)	Taichung, Taiwan	24.1199, 120.6760	*Apis mellifera*
MN857157	AM-TW(YL)	Yilan, Taiwan	24.7472, 121.7460	
MT240777	AM-TW(CT)1	Taichung, Taiwan	24.1199, 120.6760	
MT240778	AM-TW(CT)2	Taichung, Taiwan	24.1199, 120.6760	
MT240779	AM-TW(CT)3	Taichung, Taiwan	24.1199, 120.6760	

Table S3. Single-strand RNA viruses (published/under review/unpublished) discovered in the yellow crazy ant, *Anoplolepis gracilipes* (Note that all the viruses are found widespread in the ant's putative native range, but persist at low prevalence or are absent in most of the ant's known introduced ranges such as East Asia)

Family	Genus	Species	Status
	Published		
Dicistroviridae	*Triatovirus*	Anoplolepis gracilipes virus 1 (AgrV-1)	Low prevalence or absent in East Asia
Dicistroviridae	*Triatovirus*	Anoplolepis gracilipes virus 2 (AgrV-2)	Absent in East Asia
	Under review/Unpublished		
Polycipiviridae	*Sopolycivirus*	Anoplolepis gracilipes virus 3 (AgrV-3)	Low prevalence or absent in East Asia
Dicistroviridae	-	Anoplolepis gracilipes virus 4	Low prevalence or absent in East Asia
Dicistroviridae	-	Anoplolepis gracilipes virus 5	Absent in East Asia
Inflaviridae	-	Anoplolepis gracilipes virus 6	Low prevalence or absent in East Asia
Inflaviridae	-	Anoplolepis gracilipes virus 7	Low prevalence or absent in East Asia
Riboviria	-	Anoplolepis gracilipes virus 8	Absent in East Asia
